# Regulatory considerations in oncologic biosimilar drug development

**DOI:** 10.1080/19420862.2015.1040973

**Published:** 2015-05-11

**Authors:** Judith C Macdonald, Helen Hartman, Ira A Jacobs

**Affiliations:** 1 Pfizer Ltd.; Tadworth, Surrey, UK; 2 Pfizer Inc.; Collegeville, PA, USA; 3 Pfizer Inc.; New York, NY, USA

**Keywords:** regulatory, biosimilars, biologics, labeling, naming, interchangeability

## Abstract

Biosimilar monoclonal antibodies are being developed globally for patients with different types of solid tumors and hematologic malignancies. Applications for proposed biosimilar monoclonal antibodies are being submitted to the regulatory authorities around the world and may increase patient access to key treatment options upon approval. An understanding among stakeholders (e.g., physicians, patients and their caregivers, pharmacists, payers) of the approval criteria, as well as the similarities and differences in regulatory pathways involved in biosimilar approval in different countries, as presented in this review, will facilitate identification of high-quality, safe, monoclonal antibodies that have been developed according to strict, biosimilar regulatory standards. Further guidance and resolution of the ongoing discussions on biosimilar labeling, naming, automatic substitution, and indication extrapolation may ensure, in the future, an effective and appropriate use of biosimilar monoclonal antibodies by oncologists and other stakeholders in daily clinical practice.

## Abbreviations

ADR: adverse drug reaction
ASBM: Alliance for Safe Biologic Medicines
CBER: US Center for Biologics Evaluation and Research
CDER: US Center for Drug Evaluation and Research
EBE: European Biopharmaceutical Enterprises
EMA: European Medicines Agency
EPAR: European Public Assessment Report
FDA: US Food and Drug Administration
INN: International Non-proprietary Name
mAbs: monoclonal antibodies
SEB: subsequent entry biologic
SmpC: Summary of Product Characteristics
WHO: World Health Organization

## Introduction

Biosimilars are biologics that are highly similar to their reference biologic products, notwithstanding minor differences in clinically inactive components. Biosimilars are currently being used in clinical practice for the supportive care of patients with cancer, such as hematopoietic growth factors (e.g., erythropoietin, filgrastim).^1^ In the next few years, a number of biologics used for the treatment of patients with cancer will lose exclusivity, including the monoclonal antibodies (mAbs) trastuzumab, rituximab, cetuximab, and bevacizumab. This loss of exclusivity enables biosimilar mAbs to be approved by regulatory authorities, and thus enter clinical use. Awareness of these upcoming changes may be of substantial value to clinicians, pharmacists, and patients and their caregivers to ensure that lower-cost alternatives enable savings within healthcare systems.^1-6^

In this review, we provide an overview of the regulatory standards for the development and approval of biosimilars in the European Union (EU) and the United States (US), outlining some of the current differences and their potential implications, and we consider some of the challenges that arise for emerging countries in adopting the biosimilar regulatory paradigm pioneered in the EU/US. Further, we review a few of the key issues for biosimilars that are currently being discussed on a global basis by regulators, biosimilars developers, and other stakeholders. We address labeling, naming, interchangeability, potential automatic substitution, and indication extrapolation, as they may significantly impact future clinical practice in oncology and other therapeutic areas.

## Biosimilars Approval Pathways in the EU and the US

The aim of a biosimilar development program is to produce a biologic that is as similar as possible to the reference product (**Table 1**). The biosimilar regulatory paradigm rests upon being able to detect whether any differences between the biosimilar and its reference product are clinically meaningful in terms of safety, purity, and potency. The most sensitive means to do this is to leverage the advances in analytical characterization as the first step in comparing the potential biosimilar with its reference product. Any differences observed must then be further evaluated via a successive stepwise approach, including in vitro functionality comparisons and/or in vivo preclinical comparisons. Next, comparative pharmacokinetic and pharmacodynamic studies are undertaken in healthy human subjects or in patients, followed by comparative clinical efficacy and safety evaluation in the most sensitive population to detect differences. The extensive analytical and non-clinical comparisons allow the clinical efficacy studies to be more tailored and targeted in addressing whether any existing differences are clinically meaningful.

**Table 1.** Similarities and differences in EMA and FDA regulatory approval pathways

	European Medicines Agency (EMA)	United States Food and Drug Administration (FDA)
First approved biosimilar	• Omnitrope® (2006)	• Zarxio^TM^ (2015)
Biosimilar regulatory paradigm	• Demonstration that a potential biosimilar is highly similar to its reference product in safety, purity, or potency/efficacy, without clinically meaningful differences	
In vivo comparative toxicology studies	• Not required routinely as default, relies more on in vitro evaluation of structure- function relationships	• Generally routinely required although FDA has the ability to waive this
Multi-step comparison of a biosimilar to its reference product	• Analytical and functional studies • In vivo non clinical analyses • Clinical pharmacokinetic/pharmacodynamic assessments • Head-to-head clinical trials in most sensitive population(s): safety, efficacy, and immunogenicity studies	
Biosimilar review process	• Non-therapeutically aligned structure in centralized CHMP reviews	• Therapeutically aligned structure
Legal pathway	• The biosimilar pathway is a separate branch of the generic pathway (Directive 2001/83/_EC_, Article 10.4)	• Biologics Price Competition and Innovation Act (BPCI Act) of 2009
Meetings between developers/sponsors and regulatory agencies	• Centralized advice procedure by the EU CHMP Scientific Advice Working Party provides mostly written advice; meetings are called in case of disagreement with proposed plan • Advice procedures with individual EU country health authorities usually involve meetings	• FDA meeting structure defined by the Biosimilar User Fee Act (BsUFA for biosimilar applications) • Biosimilar Product Development (BPD) meetings enable Biologic License Applications under 351(k) pathway
Inter-agency meetings	• EMA and FDA cluster meetings (closed, regulators-only meetings) • EMA/FDA parallel advice (for companies)	

A helpful analogy when considering the challenge faced by a biosimilar developer is to consider it as aiming to produce a version of Leonardo Da Vinci's Mona Lisa painting, which is as close as possible or undistinguishable from the original, but created without knowing the conditions and materials that produced the original product. The biosimilar developer does not have access to the manufacturing or precise cell-line details used to create the innovator reference product and must work in reverse, by first studying the reference product to create a similar version. This requires considerable skill and expertise in the development and production of biologic products. For biologics, it is often stated that the ‘process is the product’, meaning that the process has a huge impact on the product. Given that biosimilar developers have to create their own process, there will inevitably be differences between the biosimilar and the reference product. Differences that are not clinically meaningful are allowable, but differences that are clinically meaningful may preclude further development as a biosimilar.

The EU first pioneered these principles in 2005 with the release of specific guidance for the development of biosimilars, based on the principle of a stepwise, comparative approach to establish similarity. Additional guidances/recommendations followed to ensure the safety and efficacy of biosimilar products.^7-9^ The first biosimilar product, Omnitrope® (Sandoz GmbH, Kundl, Austria) was approved in the EU in 2006. In the US, the Biologics Price Competition and Innovation Act (BPCI Act) was signed into law in 2010 as part of the Patient Protection and Affordable Care Act (PPAC Act) to provide a regulatory pathway for the development and approval of biosimilars, and thus allow greater access to biologic therapies for patients.^10^ This was followed by the release of several draft guidance documents on biosimilar products in 2012 and, more recently, the release of draft guidances on clinical pharmacology and on formal meetings between the biosimilars developers and the US Food and Drug Administration (FDA).^11–14^ Recently, the FDA has begun evaluating the first applications under the 351(k) biosimilars pathway and has just approved the first biosimilar filgrastim (Sandoz) as of 6 March 2015.^15^

The overarching principles of how biosimilars should be developed are highly aligned at a macroscopic level between the EU and the US. In both the EU and US, a biosimilar is produced by reverse engineering in such a way as to generate a version of the product that is as similar as possible to the original reference product, with no clinically meaningful differences in terms of safety, purity, and potency. Therefore, both EU and US regulations require head-to head comparative studies to demonstrate similarity with respect to structural and functional characterization, in vitro biologic assays, and pharmacokinetic and pharmacodynamic evaluations, as well as safety, efficacy, and immunogenicity studies.^11,12^

Notwithstanding the overarching regulatory alignment and the common goal of approving high-quality, safe medicines, there may be subtle differences between the EU and US agencies in how the principles are applied at a specific project level. The nuances of differing interpretations between FDA and EMA for requirements at a product level tend to be confidential in nature and specific to the company concerned. However, one high level difference is in the approach to comparative in vivo toxicology studies. The EU regulators generally do not require in vivo toxicology studies as a matter of course whereas the FDA generally does. The EU regulators outline their desire to follow a risk-based approach and leverage in vitro functional comparisons in a recent article by van Aerts.^16^

In addition to scientific differences in interpretation of requirements, the structure and operation of the US and EU regulatory agencies have advantages and disadvantages when it comes to ease of implementation of the biosimilar regulatory paradigm. For example, in the centralized EU Committee for Medicinal Products for Human Use (CHMP) reviews, members are not therapeutically specialized and the basis of the review is a peer-based process in which a Rapporteur/CHMP member and a co-rapporteur/CHMP member undertake the scientific assessment. The other CHMP members may raise points and put forth questions for consideration. The decision making is ultimately based on a vote from all the CHMP members on whether or not to approve the product. This type of review applies to all eligible products including biosimilars. Therefore, it may be intrinsically easier to reach agreements across CHMP members on extrapolation of indication and other points of the review, as consensus building and the need to find a harmonized approach across all the EU countries are part of the usual assessment process for all products in this procedure.

On the other hand, FDA works through therapeutically aligned divisions with deep knowledge of a therapy area, and it might appear more challenging to implement the biosimilar concept which is based on the “totality of data” across a variety of therapeutic areas and multiple functional disciplines. Nonetheless, the FDA has set up several specific committees to ensure that the development of the product is in line with biosimilarity standards.^10^ The FDA biosimilar specific committees include the Biosimilar Implementation Committee (BIC), the Center for Drug Evaluation and Research (CDER) Biosimilar Review Committee (BRC), and the Center for Biologics Evaluation and Research (CBER) Biosimilar Review Committee (BRC). While the BIC allows for discussion of overarching biosimilar policy issues, the BRC allows for discussion of product-specific issues.

There is also a difference in how the legislation is enacted. In the US, the legal pathway considers biosimilars as new, active substances, and therefore they need to fulfill pediatric requirements unless otherwise justified. In contrast, in the EU, in legislative terms, the biosimilar pathway (Article 10(4) of Directive 2001/83/_EC_) is in effect a separate branch of the generic pathway (Article 10(2) of Directive 2001/83/_EC_). Biosimilars are considered as products for which the standard generic criteria of producing an identical product cannot be fulfilled, as these are biologic products produced in biological systems and, therefore, additional data are needed. The fact that the regulation of biosimilars is rooted in the generic pathway seems to have influenced European Medicines Agency (EMA) thinking in determining its current policy for labeling of biosimilars (please see the labeling section in this review).

The FDA's Biosimilar User Fee Act^17^ Biosimilar Product Development meeting structure offers distinct advantages to the sponsor. For example, the fact that a discussion meeting is generally offered in the US is extremely helpful to the sponsors by providing them with the opportunity to clarify and discuss matters with the agency, thus facilitating the development of biosimilars. Additionally, the Biosimilar Product Development meetings^17^ enable a Biologic License Application–type review of raw data on chemistry, manufacturing, and controls, and on pharmacokinetics, which is out of scope for EU scientific advice.

In Europe, centralized scientific advice procedure by the EU CHMP's Scientific Advice Working Party provides a pan-EU perspective and generally does not involve a discussion meeting with the developer. There is the option for this to happen, but it is generally only invoked when the CHMP disagrees with the applicant's proposed plans. By contrast, national advice procedures with individual country health authorities in Europe virtually always involve a meeting with the regulators. In considering the complex and evolving issues related to the development of biosimilars, sponsors find meetings to be highly valuable given that it is important to meet with the regulators early in the development program and to meet more frequently than would be required for a novel product. Nonetheless, the FDA and EMA have regular biosimilars cluster meetings in which regulators can share their experiences and harmonize their thinking. In addition, several biosimilar sponsors have used EMA/FDA parallel advice as a means to resolve differing advice between FDA and EMA. Although the process still results in each agency issuing separate written advice at the end of the procedure, it is highly valuable for the sponsor to participate in the joint discussion meetings. The final written feedback is often more harmonized as result of the joint discussions than would have been likely pursuing individual consultations.

## Challenges in Emerging Countries

While there is general alignment in the biosimilar standards in the EU and US, there is greater diversity in the definition of follow-on products to originator biologics and implementation of strict biosimilar principles in emerging countries.^18^ The World Health Organization (WHO) guidance on similar biotherapeutic products,^19^ which can ensure a harmonized global regulatory standard, was largely built upon the principles of the EU guidance.^7-9^ However, regulatory agencies in emerging countries face challenges in terms of building capability and capacity to assess biologic products as a whole and, consequently, biosimilars. The WHO plays a key role in this regard in holding a number of conferences and workshops to help build awareness of these products.^20^ However, building capability and capacity takes time, and yet the clear and pressing need for access to these products is of pivotal importance.

In the meantime, a number of emerging countries, including Columbia and India, have elected to adopt alternative standards for the regulation of follow-on biologics which may not fully comply with the WHO guidance, resulting in the approval of follow-on biologics which have not been rigorously compared with the reference product at the analytical, nonclinical, or clinical level. The International Federation of Pharmaceutical Manufacturers and Associations (IFPMA) has termed these “non-comparable biotherapeutic products” to distinguish them from biosimilars developed and approved according to WHO standards.^21^ Non-comparable biotherapeutic products would not be considered biosimilars in the EU and the US, as they have not fulfilled the strict EU/US approval requirements. In their policy paper on non-comparable biotherapeutics, IFPMA outlines its concerns with this approach as “in some cases non-comparable biotherapeutic products may have little or no safety, efficacy or immunogenicity data, since they may have been brought to market using regulatory pathways designed for chemically synthesized drugs, generic medicines or similarly abbreviated approval processes, which do not require such data for licensure.”^21^ Review and approval in emerging countries of non-comparable (also termed non-innovator or intended copy) pharmaceutical products with limited, comparative data may create potential confusion for prescribers, patients, and payers, as well as increased risks for their use, especially because such products may coexist on the market with biosimilars regulated according to WHO standards.

In contrast to the views of the IFPMA, some stakeholders including the Associação Brasileira Interdisciplinar de Aids, the All India Drug Action Network, the Colegio Nacional de Químicos Farmacéuticos de Colombia, the Argentinian Fundación GEP, the Peoples' Health Movement, and the Third World Network believe that the EU/WHO/US regulatory standards for biosimilars represent too high a hurdle and an unnecessary barrier that prevents access to medicines. This is exemplified in the Civil Society statement, “Time to act to ensure access to affordable biotherapeutics,” which was presented at the 2014 Pre-International Conference of Drug Regulatory Authorities Biosimilars Conference.^22^ However, if the regulatory standards for biosimilars were set too high in the developed world, we would expect there would be very few regulatory approvals issued. Taking the EU as an example, we find that, since 2005, a total of 19 biosimilars currently hold a marketing authorization.^23^ The high number of approvals does not support the premise that the standards are too high and that there is “over-regulation” of these products. It is also important to point out that the opposite extreme—setting too low a standard for biosimilars—would also be undesirable since such “under-regulation” could potentially allow approvals that would represent a risk to public health. In the EU, there have been 8 unsuccessful biosimilar marketing authorization applications to date. This demonstrates that the EU system for biosimilar approvals, upon which the WHO guidance is based, is capable of denying approval of products that do not meet the required standards of biosimilarity. Thus, it can be concluded that the biosimilar regulatory paradigm has been effectively “road tested” in the EU and the standards have been appropriately set. Emerging countries must decide how to ensure access to follow-on biologics while setting an appropriate regulatory standard where the equally damaging extremes of over-regulation and under-regulation are avoided.

## Labeling and Extrapolation

Currently there is no global harmonized approach to biosimilar labeling. In the EU, in legislative terms, the biosimilar pathway (Article 10(4) of Directive 2001/83/_EC_) is in effect a separate branch of the generic pathway (Article 10(2) of Directive 2001/83/_EC_). Biosimilars, however, are considered products for which the standard generic criteria of producing an ‘identical’ product cannot be fulfilled, as these are biologic products produced in biological systems and, therefore, additional data are needed. The fact that the regulation of biosimilars is rooted in the generic pathway seems to have influenced European Medicines Agency (EMA) thinking in determining its current policy for labeling of biosimilars In Europe, the EMA has recently indicated that labels for biosimilars should include only information on reference products, and has elected to place all mention of the comparative clinical and preclinical data of the biosimilar in the European Public Assessment Report (EPAR) with no citation of these data in the label.^24^ The EPAR contains a summary of the review process and thus, it often contains further details of the studies undertaken with a product. In this case, as there is no mention of comparative study results in the label and it is not stated that all the information was generated with the original reference product and not with the biosimilar, physicians may not search for additional details in the EPAR, potentially assuming, incorrectly, that all the data generated on the product are already contained in the label. Although the biosimilar label does specify that the product is a biosimilar, which is helpful for physicians and patients to know, this is not mentioned upfront at the beginning of the label, but midway through the document. There could be important information related to a biosimilar product, such as comparative immunogenicity rates, which would be important for the physician to have ready access to via the label, rather than searching for this information in the EPAR. Similarly, patients should be informed about the product. Thus, it would seem appropriate to consult physicians and patients on what information they would find helpful in a biosimilar label.

Of note, in a survey recently conducted by the Alliance for Safe Biologic Medicines (ASBM) among European physicians based in France, Germany, Italy, Spain, and the UK, about half (46%) of the 470 responders acknowledged they had only a basic understanding of biosimilars.^25^ Importantly, 38% reported that they never consult an EPAR to gain additional information on a product, compared with only 19% who do so routinely. Approximately 43% of the surveyed physicians noted that they refer to EPARs only occasionally. In contrast, more than 80% of the responders reported using the Summary of Product Characteristics (SmPC) and the label to gain information on a product. Thus, since the SmPC is the primary reference source of information, it should contain all the salient information on a biosimilar product.^25^

Other agencies follow a different approach for labeling biosimilars. For example, the Swiss Medic Guidance for biosimilars recommends that information on both the reference product and the biosimilar be included in the label, and the data relevant to the biosimilar be easily identifiable.^26^ Similarly, Health Canada specifies that subsequent entry biologics (SEBs)/biosimilars should not use the product monographs of the reference biologic products in their entirety (as is the case for generic drugs). The product monographs for SEBs should indicate that the product is a SEB and present key data supporting its marketing authorization, including tables with results of the comparison between the SEB and the reference product.^27^ Such an approach has been followed by Health Canada in the recent approval of a biosimilar infliximab antibody. In Australia, the Therapeutic Goods Administration mandates compliance for biosimilars product information and consumer medicine information with the general requirements for medicine labels.^28^ Clinical trial information on the reference product may be included, but it should be clearly identified as having been obtained with the originator and not with the biosimilar.^28^

The European Biopharmaceutical Enterprises (EBE) Biosimilars Task Force, a specialized group of the European Federation of the Pharmaceutical Industries and Associations, has recommended in its position statement that specific information on both the reference product and the biosimilar should be included in the label to ensure transparency, as biosimilars are not identical to their reference products. Furthermore, traceability and appropriate safety reporting are very important for biologics, including biosimilars.^29^ In addition, this group has recently published an article explaining why a transparent and complete approach to product labeling is an important tool in building patient and physician confidence in biosimilars.^30^

In the US, the labeling for the first FDA-approved biosimilar, Zarxio, is similar in structure and content to the labeling used for generic small molecules.^15^ The Zarxio label does not indicate that the product was approved as a biosimilar nor does the label contain any data specific to Zarxio's demonstration of biosimilarity. The FDA noted that the “approach we took for this label is not that different from the approach we have taken in the past for other situations, such as for generic applications and 505(b)(2) applications” and that the information in the Zarxio label is sufficient “for a prescriber to make an appropriate prescribing decision for their patient”.^15^ According to the FDA, additional specific guidance on labeling of biosimilars is forthcoming.^31^ To ensure transparency, label updates are currently being discussed for pharmaceuticals in the US, following release of the 2013 FDA Proposed Rule change,^32^ which may lead to label differentiation. According to this FDA Proposed Rule, safety-related updates, based on post-marketing pharmacovigilance reports, may yield information that should be added to specific product labels, thus resulting in labeling changes for generic drugs, independently of the originator's or other generic drug labels.^32^ It is currently unclear whether such a Proposed Rule on label safety updates may have potential future implications for labeling biosimilars in the US.

Extrapolation of indications is a topic that is actively debated within the oncology community, as it represents an important issue for future approval and labeling of antineoplastic biosimilar mAbs, such a trastuzumab, rituximab, cetuximab, and bevacizumab, whose reference products are currently indicated for multiple disease stages across different tumor types.^1,2,33^ Extrapolation is allowed by both the EMA and FDA for biosimilars, although this must be based on a strong scientific justification, which leverages the complete comparability data set (quality, non-clinical and clinical evidence, as well as what is known on the mechanism of action [MOA]).^34^ Of note, the EMA has recently allowed extrapolation of indication for rheumatologic and gastrointestinal inflammatory diseases at approval of the 2 infliximab biosimilar mAbs, Inflectra™ (Hospira UK Ltd, Royal Lemington Spa, UK) and Remsima™ (Celltrion Healthcare Hungary Kft, Budapest, Hungary).^35,36^ In contrast, Health Canada did not agree that extrapolation to the inflammatory bowel disease indications (e.g., Crohn's disease and ulcerative colitis) was justified for Remsima.^37^

From a regulatory perspective, clinical trials should not be needed for every indication in oncology once the comparative, stepwise assessment has demonstrated biosimilarity to the reference product without clinically meaningful differences, provided that a strong scientific justification can be made.^7,8,11,12^ The scientific justification for extrapolation of the safety and efficacy findings from the patient population that was studied in the biosimilar clinical trial to a different population should address differences in the safety, risk profile, and comorbidities between the populations, differences in concomitant medications, and differences in the MOA. In other words, biosimilar developers need to provide sufficient scientific evidence to allow extrapolation of available data “to support a determination of biosimilarity for each condition of use for which licensure is sought”, as indicated by the FDA in its Scientific Considerations in Demonstrating Biosimilarity to a Reference Product guidance for biosimilars.^11^ Accordingly, evidence of comparability in terms of target/receptor for each product's activity, patterns of product/target interaction (including binding, dose response, and molecular signaling), and target/receptor localization may support demonstration of biosimilarity in MOA. Further, pharmacokinetic and biodistribution analyses, as well as pharmacodynamic assessments may provide additional evidence of a comparable MOA and activity for the biosimilar product across different patient populations. Information should also be provided on potential differences in safety profile and adverse events expected in each indication and patient population under consideration.^7,11^ Ultimately, appropriate comparative, clinical evaluation of biosimilar mAbs in the most sensitive patient populations, supported by relevant scientific data on MOA, if available, may provide the body of evidence needed to approve the full range of indications for the use of antineoplastic biosimilars in patients with cancer.

Identification of the most sensitive patient population(s) for comparative clinical evaluation of specific biosimilars is currently being debated across tumor types (eg, neoadjuvant vs. metastatic setting for trastuzumab) and specialty (eg, oncology vs. rheumatology for rituximab). In breast cancer, determination of the total pathologic complete response in the neoadjuvant setting, rather than overall response rates in patients with metastatic disease may represent a sensitive setting for demonstration of biosimilarity in efficacy for trastuzumab biosimilars. Early breast cancer may also provide an appropriate setting for comparative evaluation of immunogenicity. The clinical data generated will support extrapolation arguments.^33^ However, it should be noted that extrapolation depends on the totality of evidence and not on clinical data alone. In conclusion, it is clear that extrapolation is a complex area, as different regulatory agencies may arrive at different conclusions on whether extrapolation of indications is justified for a given biosimilar, and decisions are expected on a case-by-case basis.^34^

## International Nonproprietary Names and Naming of Biosimilars

Although the WHO International Non-proprietary Names (INN) system, first adopted in the 1950s, defines the global standards for the nomenclature of pharmaceuticals, naming of biosimilars is still unresolved and actively discussed by stakeholders in many regions, including the US.^38-42^ The Generic Pharmaceuticals Association and other associations are supporting use of the same innovator INNs for biosimilars as used for the reference product, a policy that is currently accepted for generic drugs. They believe that a brand name provides adequate identification for the product in terms of tracing adverse drug reactions (ADRs) for the product concerned.^40^ Conversely, in view of their complexity and potential microheterogeneity, the Pharmaceutical Research and Manufacturers of America, the Biotechnology Industry Organization, the EBE, and the ASBM support the adoption and use of distinguishable, international, non-proprietary names for biosimilars as an additional means of identification beyond use of the invented brand name, as it is not a mandatory requirement for products to have an invented proprietary brand name in either the EU or the US.^39,41,42^

Naming of biosimilar products has important implications for physicians' prescription, potential patient bias, and interchangeability, as well as pharmacovigilance. Each biosimilar product should be readily distinguishable from the reference product and other biosimilars to ensure appropriate use, traceability, and accurate reporting of ADRs.^43^

Different countries, such as Japan, have adopted their own policies for biosimilar naming. Currently in Japan, the non-proprietary and proprietary names of biosimilars should be easily distinguishable from those of other biosimilars and those of the reference products, according to the guidance released by the Pharmaceuticals and Medical Devices Agency, the Japanese regulatory agency.^44^ Non-proprietary names of biosimilars should contain, at the end of the name, the respective follow-on number (e.g., biosimilar 1, 2, or 3), and the proprietary name should contain the BS letters, in addition to the dosage form, dosage, and name of the manufacturer.^44^

In other regions, particularly in emerging markets, the lack of specific regulatory guidance for development/naming of follow-on biologics or the existence of different approval pathways currently represents a substantial challenge for stakeholders, including clinicians, pharmacists, and patients, as well as their caregivers, who may be confronted, as a consequence, with the approval and use of ‘intended copies' or non-comparable versions of biologic products. Specifically, in certain countries, such as Brazil, China, Columbia, India, and Mexico, local regulators have approved biologics that are intended to be a copy of the innovator (“intended copies”), even though the development was not conducted in a rigorous, stepwise comparison with the reference product, according to EU/US guidance and WHO recommendations. Reports of safety issues with some of these products, such as recombinant erythropoietins, have raised concerns about the quality of these biologics.^45-48^ For example, increased rates of pure red-cell aplasia were observed in patients with chronic kidney disease who received subcutaneous administration of recombinant human erythropoietin compared with the reference product. Such adverse reaction appeared associated with an increased immunogenicity of the biologic product, with induction of anti-erythropoietin antibodies and pure red-cell aplasia in treated patients.^48^ The term biosimilar should, therefore, be reserved for products developed in a comparative fashion, according to strict regulatory standards.^7,8,11,12^

The fact that these copy biologics often share the same INNs as the reference products underscores why having an INN qualifier unique to the manufacturer would be advisable from a traceability and patient-safety perspective. As indicated by the WHO, national regulatory agencies need to ensure accurate ADR reporting for marketed biopharmaceuticals, by requiring inclusion of proprietary (brand) name, manufacturer's name, lot number, and country of origin, in addition to the INN.^49^ However, ADR-reporting issues may be compounded in case of incomplete or inaccurate information, because innovator companies generally have a policy of accepting ADR reports as belonging to their product if they cannot reasonably exclude an association with the use of their own product. Thus, a true signal on a biosimilar may actually be missed by being included in the originator's safety database, and thereby diluted by other reports in the system. In addition, if the pharmacovigilance system in a given country is not implemented based on high regulatory and efficiency standards, it may not be possible to detect safety signals related to specific intended-copy products. Finally these difficulties may be compounded when a country introduces specific and rigorous regulatory guidance for biosimilars and intended copies are already available on the market and prescribed to patients. To address this issue, the WHO is currently developing guidance on a risk-based approach.^50^

The WHO has recently acknowledged the need to distinguish biosimilar products. In August 2014, it released a draft proposal for adding a 4-letter code, or “biological qualifier” to all biologics, in addition to the INN, to establish a more robust system for the identification of biosimilars and other biologics.^51^ While adoption of the biological-qualifier system would be a voluntary decision by individual regulatory authorities, it would represent an important tool for global harmonization.

## Interchangeability and Automatic Substitution

Both similarities and differences exist between the US and the EU perspectives on generic drug substitution and interchangeability of biosimilar. In the US, the Approved Drug Products with Therapeutic Equivalence Evaluations publication (“Orange Book”), which is constantly updated by the FDA, provides a list of conventional drugs with demonstrated therapeutic equivalence, but it does not mandate specific generic substitutions.^52^ In the absence of specific federal regulations, state legislations in the US play a substantial role in determining patient access to approved generic drugs. State laws may require physician notification of substitution of a brand product with a generic drug.^53^ Similarly, substitution of generic drugs considered equivalent to the reference products may be applied in European countries for many non-biologic pharmaceuticals, although the policies vary from country to country.

For biosimilars, the US is the only country which currently allows a formal ‘interchangeable’ designation for biologic products. Specifically, the FDA Biologics Price Competition and Innovation Act of 2009 envisions interchangeability, provided that manufacturer's study results demonstrate that a biologic agent is “biosimilar to the reference product” and “can be expected to produce the same clinical result as the reference product in any given patient.”^10^ Further considerations are included aimed at ensuring patient safety for chronically administered products when switching biologic therapy. However, the FDA has not yet released specific recommendations regarding the scientific standard required for interchangeability, although it is clear that a higher standard applies, beyond approval of the product as a biosimilar. The CDER list of guidelines under development in 2014 includes an interchangeability guidance on “considerations in demonstrating interchangeability to a reference product”; however, the exact timing when this might be issued is unclear.^31^ In September 2014, the FDA released the first edition of the Lists of Licensed Biological Products with Reference Product Exclusivity and Biosimilarity or Interchangeability Evaluations (“Purple Book”), which will provide periodically updated information for regulators, manufacturers, and clinicians on biologic product exclusivity and biosimilars.^54^ In the absence of specific federal guidance, states in the US have started to independently legislate on biosimilar substitution, and may continue to do so, even if biosimilars are deemed interchangeable by the FDA. Elements that may be common to such legislations include interchangeability only if the FDA has recommended such designation for a specific biologic product, as well as the need for patient and/or prescriber notification by pharmacists and appropriate record keeping in case of a substitution.

Interchangeability and substitution of biosimilars are not within scope of EU regulatory approval and, hence, there is no agreed upon definition of what interchangeability actually means and no inclusion of such information in the EPAR.^24^ EU regulators believe that biosimilars are “therapeutic alternatives” to the reference product, which would allow a biosimilar to be switched for the reference product either at initiation or during therapy.^6,34^ However, the European Consensus document released by the European Commission notes that interchangeability implies an initiative or agreement by the prescriber, and that patients should speak to their physician and pharmacist about switching decisions and changing therapy from one biologic product to another.^24^ Consistently, the majority of the European physicians participating in the ASBM survey agreed that they should retain sole authority in the substitution process, specifying that they found it “critically important” (24% of responders), “very important” (48%), and “somewhat important” (23%) to retain such authority.^25^ Potential issues related to the safety and efficacy of biosimilars including immunogenicity (eg, due to the switch between different products), incidence of potential adverse reactions, and specific activity over time are related to the complexity of these biologic drugs and their production in biologic systems. Approval of biosimilars following strict EMA/FDA standards, production according to Good Manufacturing Practices, reliable supply chains, careful post-marketing surveillance, and clinical experience in their use may all contribute to build a strong foundation for a safe and effective substitution of reference products with biosimilars.

Automatic drug substitution is the decision to switch a product for another product at the pharmacy level without the consent of the prescribing physician. Automatic substitution is generally confined to true generic drugs, which are chemically derived products that can be identical to their reference product in terms of chemical composition. Specific implementation of automatic drug substitution is independently regulated by each European country.^55^ The majority of the EU member-states do not currently allow automatic substitution of biosimilars or have excluded biologics from the official substitution lists. Conversely, France may, in the future, allow substitution by retail pharmacies of biosimilar versions of biologics upon initiation of therapy, as recently indicated in the new Social Security Budget Legislation (article 47), subject to further approval.^56^ In this case, substitution would apply to agents included in “similar biologic groups” and it would be possible only at the start of treatment, provided that a clinician has not added “non-substitutable” to the prescription.^56^

## Implications for Oncology and Conclusions

The approval of biosimilar mAbs is expected to allow increased patient access to treatment, particularly in oncology where a number of biologics are key components of standard-of-care regimens.^57^ Further guidance by the regulatory authorities in the US and the EU, with clarification and resolution of labeling, naming, and interchangeability issues for biosimilars may support further development by manufacturers, facilitate approval and integration of biosimilars in the treatment algorithms, and lead to an appropriate and safe use in daily clinical practice.

In conclusion, specific regulatory guidance and pre/post-approval education of all stakeholders, including clinicians, pharmacists, patients, and payers, may help to ensure an effective, future utilization of mAb biosimilars, long-term monitoring of their safety, and optimal patient benefit.
